# ADSCs attenuate Liver fibrosis via inducing HSC senescence: validation in dual-etiology models

**DOI:** 10.1371/journal.pntd.0013094

**Published:** 2025-05-22

**Authors:** Mukexina Mulati, Ning Yang, Junlong Xue, Liang Li, Xue Zhang, Hui Liu, Jin Chu, Guodong Lü, Nuerbaiti Kusuman, Xiaolong He, Tuerganaili Aji, Xiaojuan Bi, Renyong Lin

**Affiliations:** 1 State Key Laboratory of Pathogenesis, Prevention, and Treatment of High Incidence Diseases in Central Asia, Clinical Medical Research Institute, The First Affiliated Hospital of Xinjiang Medical University, Urumqi, China; 2 Department of Hepatobiliary and Hydatid Diseases, Digestive and Vascular Surgery Center, The First Affiliated Hospital of Xinjiang Medical University, Urumqi, China; Institute of Cytology and Genetics SB RAS: FIC Institut citologii i genetiki Sibirskogo otdelenia Rossijskoj akademii nauk, RUSSIAN FEDERATION

## Abstract

**Background:**

Liver fibrosis (LF) results from various causes, which require finding conserved mechanisms to help treat related diseases. Although adipose-derived stem cells (ADSCs) transplantation can alleviate hepatic fibrosis, their mechanism remains unclear. Accordingly, we explored the efficacy and mechanisms behind ADSCs transplantation in two LF models.

**Methodology:**

The carbon tetrachloride (CCl_4_)-induced liver injury and *Echinococcus multilocularis* (*E. multilocularis*) infection models were established, and ADSCs were transplanted. Mouse liver samples were harvested and analyzed histologically. Expression levels of fibrosis and senescence-related proteins were analyzed by immunohistochemistry. Hepatic stellate cells (HSCs) activation and cell senescence protein expression were evaluated via western blotting. Co-localization expression was determined by immunofluorescence. To assess the cellular senescence degree, we utilized senescence-associated β-galactosidase (SA-β-Gal) staining.

**Result:**

In the CCl_4_-induced LF mouse model, the liver surface exhibited a rough texture. The hematoxylin and eosin (H&E) staining revealed hepatic parenchymal cell destruction accompanied by pseudolobule formation and fibrosis in the portal area. In the *E. multilocularis* infection model, multiple white foci were on the liver surface. The H&E staining revealed massive inflammatory cell infiltration around the foci with severe fibrosis, and Sirius Red confirmed collagen deposition; both had elevated HSCs activation (α-SMA expression). After ADSCs transplantation, the collagen deposition and HSCs activation significantly diminished, while the cellular senescence levels increased. Immunofluorescence co-localization of p21 and a-SMA showed that transplantation of ADSCs promoted senescence of activated HSCs (aHSCs). *In vitro* co-culture had similar results, accompanied by expression changes and TGF-β/Smad signaling inhibition.

**Conclusion:**

Our findings indicate that ADSCs transplantation can mitigate fibrosis by inducing the senescence of aHSCs and reducing collagen production.

## Author summary

Liver fibrosis (LF) represents a grave pathological condition with the potential to progress to liver failure. It stems from diverse etiologies, encompassing chemical injury and parasitic infestations. In our investigation, we delved into the therapeutic implications of transplanting adipose-derived stem cells (ADSCs) in the management of LF. By employing two distinct mouse models—one induced by carbon tetrachloride (CCl_4_) and another by *Echinococcus multilocularis* infection—we observed a marked decrease in liver scarring and collagen accumulation following ADSCs transplantation. Notably, our research uncovered a mechanism by which ADSCs exert their beneficial effects: they induce senescence in activated hepatic stellate cells (aHSCs), the primary drivers of fibrosis. Senescence in aHSCs reduces collagen synthesis, thereby facilitating the reversal of fibrosis. The present study enriches the understanding of ADSCs-mediated antifibrotic mechanisms and holds promise for the development of novel therapies to improve clinical outcomes for patients with fibrosis-related diseases.

## Introduction

Liver fibrosis (LF), a critical pathological process in chronic liver disease progression, is defined by the scarring reaction resulting from a persistent wound-healing process within the liver. This process can compromise the normal architecture and functionality of the liver, thereby adversely affecting its diverse functions, including metabolism, detoxification, synthesis, and secretion [1]. The etiology of LF includes a myriad of factors, such as viral infections [2], long-term and heavy alcohol consumption [3], chemical damage (e.g., caused by carbon tetrachloride, CCl_4_) [4], and parasitic infections (e.g., caused by *E. multilocularis* infection) [5].

The activation of hepatic stellate cells (HSCs), transitioning from a quiescent to an activated state in response to inflammatory signals, is a crucial first event in LF progression [6]. The proliferation of activated HSCs (aHSCs) leads to excessive accumulation of extracellular matrix (ECM) components, ultimately exacerbating LF progression [7]. Currently, LF can be mitigated by reversing HSCs activation by inducing apoptosis and senescence in aHSCs [8]. Prior research has shown that the death of aHSCs significantly inhibits the progression of LF [9,10]. Recently, aHSCs senescence has been indicated to alleviate LF development [11,12] Upon undergoing senescence, aHSCs cease proliferation and alter their secretory patterns, reducing ECM component production while increasing ECM-degrading enzyme secretion. This shift leads to the decreased synthesis and accelerated degradation of collagen, thereby facilitating LF reversal [13]. Accordingly, aHSCs induction may emerge as a superior therapeutic approach for addressing LF.

Cellular senescence is characterized by the irreversible cessation of the cell cycle. This arrested condition occurs following a constrained number of cell divisions, throughout which the cells forfeit their potential to proliferate further while retaining a level of viability as well as metabolic activity [14]. Senescent cells exhibit a large, flattened morphology and an escalated senescence-associated SA-β-Gal activity [15]. Presently, multiple drugs have been used to mitigate LF by triggering aHSCs senescence [16–18]. As important regulatory molecules in cellular senescence, the deletion of p53 or p16/Rb can lead to increased fibrosis, suggesting senescence is a protective role for fibrosis [19]. Furthermore, the extensive interactions between senescent cells and the tissue microenvironment may also be involved in limiting fibrosis progression post-hepatic injury by cellular senescence [20].

Mesenchymal stem cells (MSCs) have become a prominent therapeutic tool in liver disease management, showcasing remarkable advancements. Their inherent multipotent differentiation potential, immunomodulatory attributes, and antifibrotic capacities are instrumental in fostering liver repair and regeneration [21,22]. In combating LF, MSCs diminish the activation of HSCs by alleviating inflammatory responses [23] and further reduce the accumulation of ECM by triggering apoptosis of aHSCs via the secretion of specific cytokines [24] or intercellular communication [25]. Our lab recently revealed that the transplantation of ADSCs may mitigate *E. multilocularis* infection-generated LF through the modulation of the TGF-β/Smad7 pathway activities [26]. However, the underlying antifibrotic mechanisms of ADSCs warrant further investigation.

The present research intends to analyze the therapeutic benefits of ADSCs transplantation on different LF models as well as initially determine if ADSCs alleviate LF by influencing the senescence of aHSCs.

## Result

### Distinct pathological characteristics and therapeutic effects of ADSCs transplantation in LF induced by chemical injury and parasitic infection

**Fig 1:**
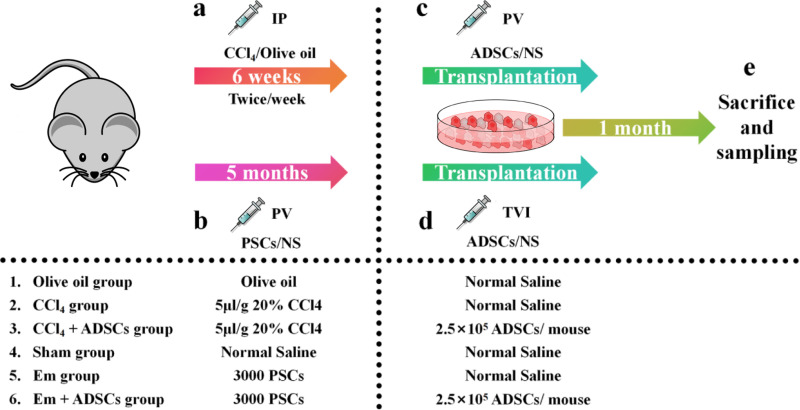
Schematic diagram of animal experimental protocol. (a) Modelling of CCl_4_ by intraperitoneal injection (IP). (b) Establishment of the *E. multilocularis* infection model by portal vein injection (PV) of protoscoleces (PSCs). (c) ADSCs transplanted via the hepatic portal vein six weeks after modeling. (d) Tail vein injection (TVI) of ADSCs five months after modeling. (e) Mice were sacrificed and sampled one month after ADSC transplantation.

**Fig 2:**
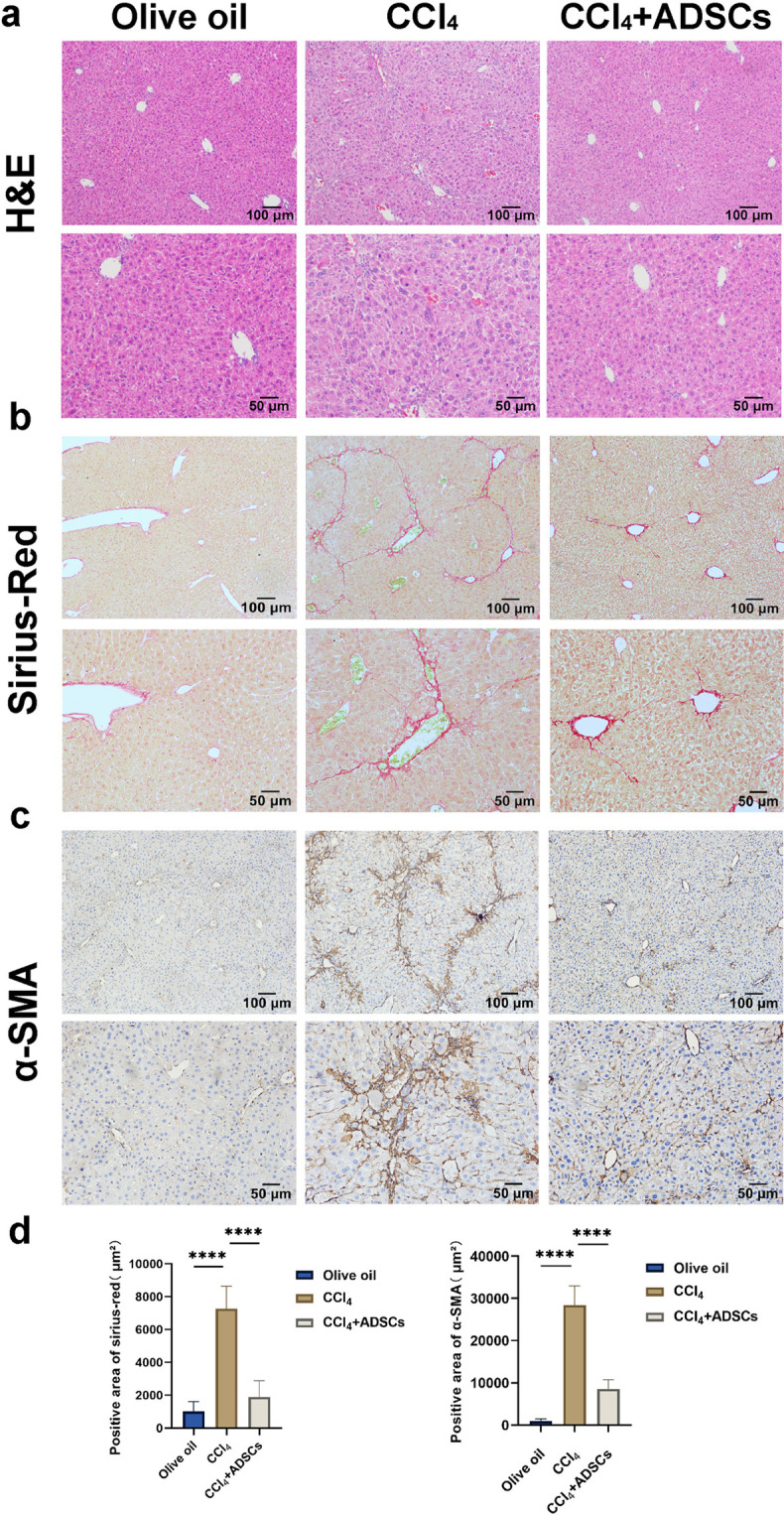
ADSCs transplantation alleviates CCl_4_-induced LF in mice. (a) Illustration of H&E-stained liver sections. (b) Illustration of Sirius Red-stained liver sections. (c) Illustration of immunohistochemical staining for α-SMA. (d) Quantitative analysis of Sirius Red and α-SMA staining across groups. Data represent mean ± SD; Statistical significance: ****p < 0.0001. Olive oil: Olive oil group (Control group); CCl_4_: CCl_4_ group (Model group); CCl_4_ + ADSCs: CCl_4_ + ADSCs group (Treatment group).

**Fig 3:**
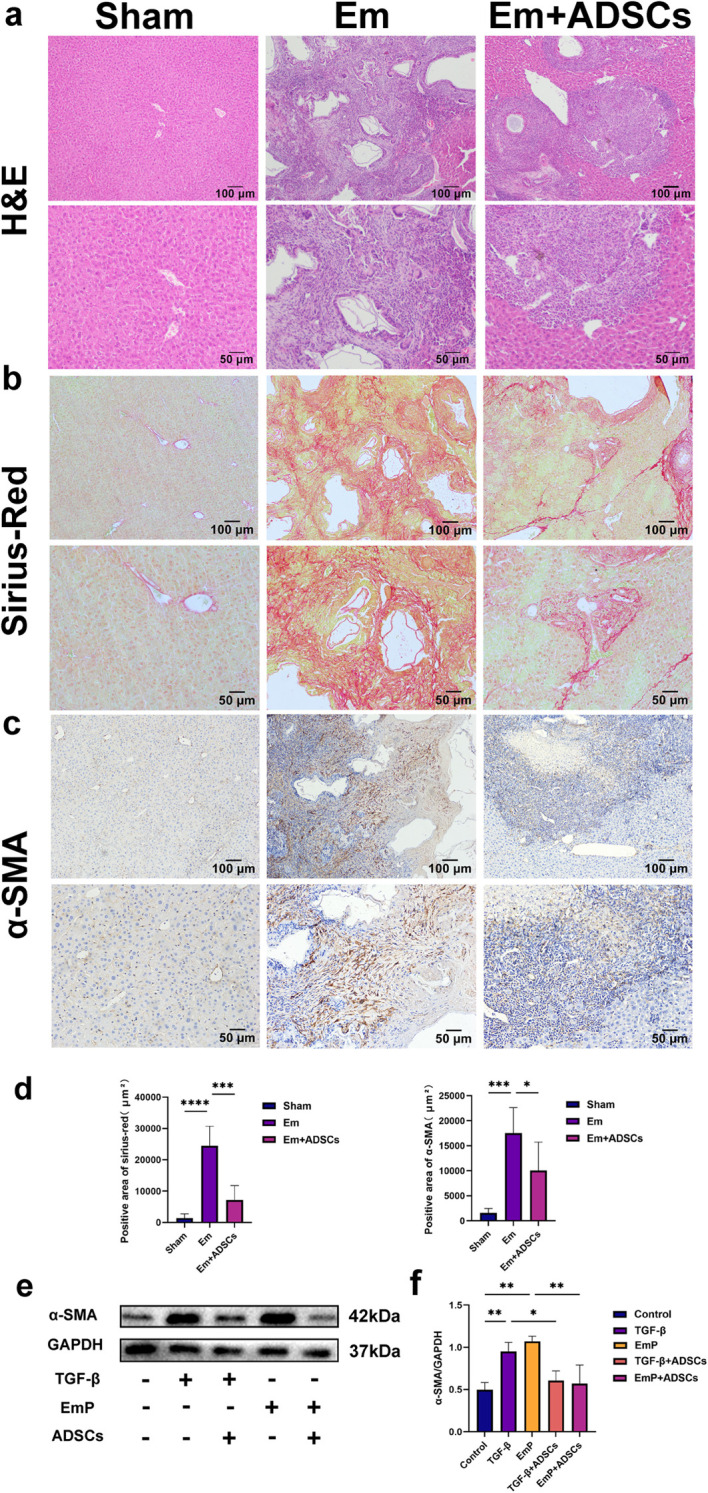
ADSCs Transplantation Alleviates Em-Induced Liver Fibrosis in Mice. (a) Illustration (a) Illustration of H&E-stained liver sections.(b) Illustration of Sirius Red-stained liver sections.(c) Illustration of immunohistochemical staining for α-SMA.(d) Quantitative analysis of Sirius Red and α-SMA staining across groups.(e-f) Quantitative analysis of α-SMA expression based on Western blotting in each group. Data represent mean ± SD; Statistical significance: p < 0.05, *p < 0.05, **p < 0.01, ***p < 0.001, ****p < 0.0001. For i*n vivo* experiment, Sham: Sham group (Control group); Em: Em group (*E. multilocularis* infection model group); Em + ADSCs: Em + ADSCs group (Treatment group). For i*n vitro* experiment, Control: Control group, TGF-β: TGF-β group (Model group), EmP: EmP group (Model group), TGF-β + ADSCs: TGF-β + ADSCs (Co-culture group); EmP + ADSCs: EmP + ADSCs group (Co-culture group).

To investigate potential shared mechanisms and therapeutic strategies for LF induced by chemical injury and parasitic infection, we established CCl_4_-induced and *E. multilocularis* infection-induced LF models (**Figs 1 and** S1). Pathological analysis revealed distinct features: CCl_4_-induced LF exhibited irregular hepatic lobules, pseudolobule formation, and portal inflammation, while *E. multilocularis*-induced LF showed localized lesions with granuloma formation and severe fibrosis (Figs 2a **and** 3a). Sirius Red (Figs 2b, 2d, 3b**, and** 3d) and α-SMA staining (Figs 2c, 2d, 3c**, and** 3d) confirmed collagen deposition and HSCs activation in both models. ADSCs transplantation significantly attenuated fibrosis in both models, reducing liver surface roughness, collagen deposition (Figs 2b, 2d, 3b**, and** 3d), and HSCs activation (Figs 2c, 2d, 3c**, and** 3d), while improving liver function in the CCl_4_ model (S2 Fig). *In vitro*, ADSCs inhibited TGF-β- and EmP-induced HSCs activation (Fig 3e) These findings demonstrate that ADSCs transplantation effectively mitigates LF by suppressing HSCs activation and collagen deposition, regardless of the underlying etiology.

### Transplantation of ADSCs can induce aHSCs senescence in both fibrosis models

**Fig 4:**
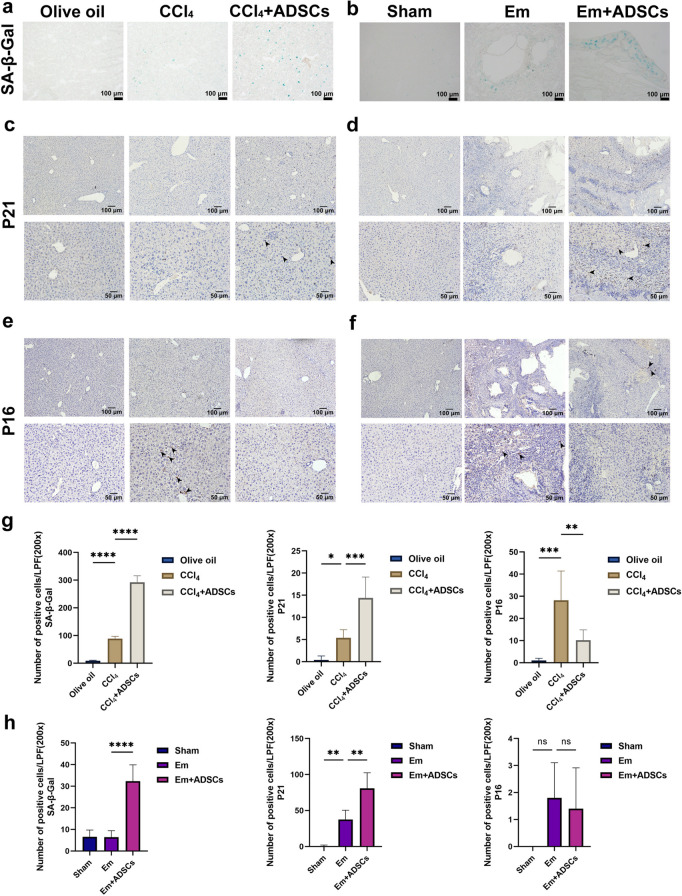
ADSCs transplantation can induce cellular senescence in mice with liver fibrosis triggered by diverse etiologies. (a-b) Illustration of SA-β-Gal staining of liver sections. (c-d) Illustration of immunohistochemical staining of p16 and (e-f) p21 of liver sections. (g-h) Quantification results of immunohistochemical staining in each group. Data represent mean ± SD; Statistical significance: p < 0.05, *p < 0.05, **p < 0.01, ***p < 0.001, *** p < 0.0001. Olive oil: Olive oil group (Control group); CCl_4_: CCl_4_ group (Model group); CCl_4_ + ADSCs: CCl_4_ + ADSCs group (Treatment group); Sham: Sham group (Control group); Em: Em group (*E. multilocularis* infection model group); Em + ADSCs: Em + ADSCs group (Treatment group). (c-d).

For evaluating the impact of ADSCs transplantation on cellular senescence in LF, we examined senescence markers p21/p16 expression in mouse livers and performed SA-β-Gal staining [27,28], revealing an increase in senescent cells in the CCl_4_ group in comparison to the Olive oil group. After ADSCs transplantation, the number of senescent cells in the CCl_4_ + ADSCs group showed a further significant increase (Fig 4a). In the Em group, the number of senescent cells was similar to that of the Sham group, with no statistical difference. Consistent with the findings in the CCl_4 _+ ADSCs group, senescent cells were significantly increased in the Em + ADSCs group (Fig 4b). Consistent with these results, immunohistochemical results showed that the senescence marker p21 expression and SA-β-Gal staining results showed the same trend in both fibrosis models, both indicating that ADSCs transplantation can induce cellular senescence (Fig 4c, 4d, 4g**, and** 4h). Interestingly, the expression of p16, another marker of cellular senescence, exhibited differences between the two fibrosis models. The p16 seemed to be predominantly expressed in the CCl_4_-induced LF model, where its expression was upregulated in the CCl_4_ group and decreased following ADSCs transplantation (Fig 4e **and** 4g). However, in the LF model induced by *E. multilocularis* infection, p16 expression was nearly absent (Fig 4f **and** 4h). This suggests that different pathways and markers may contribute to the mechanisms behind cellular senescence in these two models of fibrosis.

**Fig 5:**
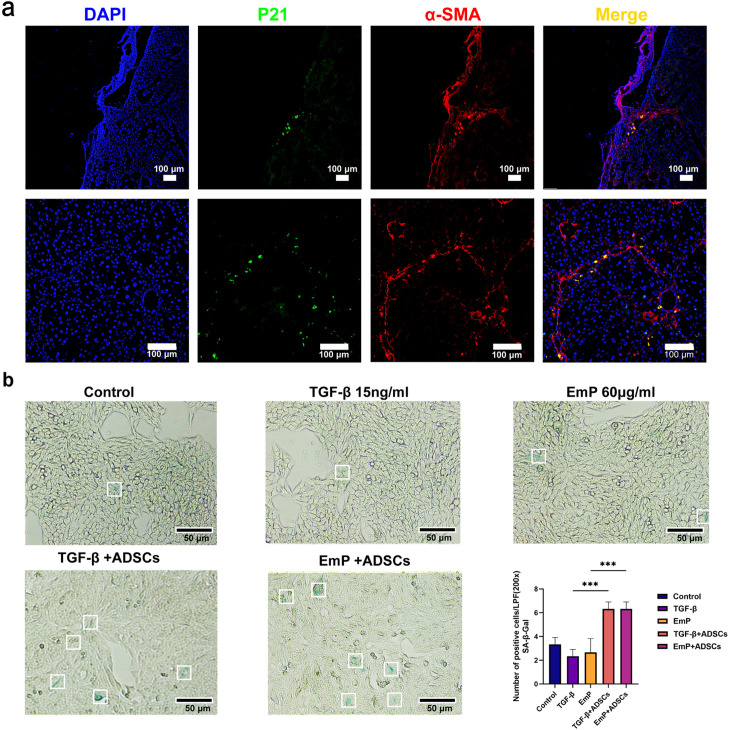
ADSCs transplantation induces senescence of aHSCs in two distinct liver fibrosis models. (a) Immunofluorescence co-localization of p21 and α-SMA after transplantation of ADSCs in two liver fibrosis models. (b) Illustration of SA-β-Gal staining of JS1 cells. Data represent mean ± SD; Statistical significance: p < 0.05: ***p < 0.001. *In vitro* experiment was conducted with three replicates. Control: Control group, TGF-β: TGF-β group (Model group), EmP: EmP group (Model group), TGF-β + ADSCs: TGF-β + ADSCs (Co-culture group); EmP + ADSCs: EmP + ADSCs group (Co-culture group).

To gain a deeper understanding of which cell populations undergo senescence following ADSCs transplantation, we conducted immunofluorescence co-localization analysis to determine the co-expression of the HSC marker α-SMA and the senescence marker p21. The immunofluorescence co-localization results showed that transplantation of ADSCs promoted the senescence of aHSCs in both fibrosis models, with p21-positive HSCs observed in distinct pathological regions: surrounding the lesions in *E. multilocularis*-infected mice and pseudolobular areas formed by fibrotic septa in CCl_4_-induced LF (Fig 5a). Consistent with the *in vivo* experimental results, SA-β-Gal staining also demonstrated that ADSCs could induce senescence in aHSCs when co-cultured with JS1 cells *in vitro* (Fig 5b). Collectively, ADSCs transplantation may exert its antifibrotic effects, at least partially, by inducing senescence in aHSCs, thereby inhibiting their proliferation and reducing collagen production, which are key events in the progression of LF.

### ADSCs promote aHSCs senescence via the p53/p21 signaling pathway

**Fig 6:**
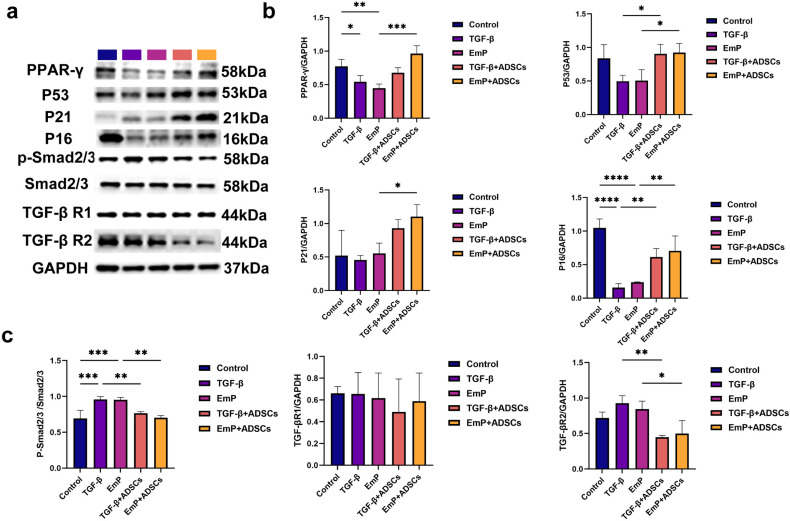
Co-culture with ADSCs induces aHSCs senescence *in vitro.* (a) Representative Western Blotting’ results for each group. (b) Quantitative results of Western blotting of senescence-related molecules and (c) fibrosis-related molecules in each group. Data represent mean ± SD; Statistical significance: p < 0.05: *P < 0.05, **P < 0.01, ***P < 0.001. The experiment was conducted with three replicates. Control: Control group, TGF-β: TGF-β group (Model group), EmP: EmP group (Model group), TGF-β + ADSCs: TGF-β + ADSCs (Co-culture group); EmP + ADSCs: EmP + ADSCs group (Co-culture group).

To mechanistically reconfirm the stimulatory effect of ADSCs on aHSCs senescence, we additionally investigated the alterations in the expression of senescence- and fibrosis-related signaling pathways in JS1 cells following co-culture with ADSCs. Western blotting results showed that co-culture with ADSCs increased the expression of senescence-associated molecules such as PPAR-γ and p53/p16/p21 in JS1 cells, promoting their senescence (Fig 6a **and** 6b). These findings are consistent with the SA-β-Gal staining results, further confirming that ADSCs can induce the senescence of aHSCs. Additionally, both TGF-β and EmP promoted the expression of fibrosis-related molecules TGF-β receptors and Smad2/3 phosphorylation in JS1 cells. However, after co-culture with ADSCs, TGF-β receptor expression and Smad2/3 phosphorylation level in JS1 cells were significantly reduced (Fig 6a **and** 6c).

## Discussion

LF is a pathological condition triggered by various insults to the liver, ranging from mild scar formation to severe cirrhosis, ultimately leading to liver failure and necessitating liver transplantation [7]. The etiological diversity of LF, including viral infections, chronic alcohol consumption, chemical exposure, and parasitic infections, underscores the complexity of developing effective therapeutic strategies [29]. Therefore, uncovering the common conserved mechanisms underlying fibrosis progression across different etiologies is crucial for the development of pan-fibrotic therapies. Addressing this critical scientific question, the present study systematically explored the therapeutic potential and mechanisms of ADSCs transplantation by establishing two distinct LF models: a CCl_4_-induced chemical liver injury model and an *E. multilocularis* infection-induced parasitic liver injury model. Both models recapitulate core pathological features of LF, including collagen deposition, inflammatory infiltration, and HSCs activation, and share conserved signaling pathways such as TGF-β/Smad and NF-κB. However, they exhibit distinct injury mechanisms and pathological phenotypes. The CCl_4_ model rapidly induces acute fibrosis within 4–6 weeks through direct chemical toxicity causing hepatocyte necrosis, with partial reversibility upon withdrawal. In contrast, *E. multilocularis* infection triggers progressive damage via parasitic infiltration, immune evasion, and chronic granulomatous reactions, forming parasite-centric “fibrotic encapsulations” surrounded by an “inflammatory-fibrotic belt,” resembling the ductal fibrosis observed in schistosomiasis. This unique spatial pattern reflects parasite-mediated modulation of host immune-fibrotic networks. Notably, the parasitic model exhibits a Th2-polarized immune microenvironment dominated by regulatory T-cell infiltration and chronic progression over months, closely mimicking human alveolar echinococcosis. Importantly, despite these differences, HSCs activation remains central to fibrogenesis in both models. Our study specifically focuses on the divergent HSCs activation states and their senescence dynamics following ADSCs intervention. By demonstrating the ability of ADSCs to mitigate fibrosis in both models, we provide compelling evidence supporting the therapeutic potential of stem cells in LF. More importantly, our research elucidated for the first time the key molecular mechanism by which ADSCs mediate fibrosis regression through inducing senescence in aHSCs. Our findings offer novel insights into the intricate interconnection between stem cells and the fibrotic microenvironment.

The HSCs, widely recognized as the primary producers of ECM in LF, undergo activation and differentiate into fibrogenic myofibroblasts, driving fibrosis progression. In contrast, senescent HSCs exhibit reduced ECM production and contribute to fibrosis reversal and recovery [30]. Recently, numerous studies have demonstrated that senescence induction in aHSCs can serve as an effective means to mitigate LF. For instance, Chen et al. [31] reported that Sjp40 can inhibit type I collagen production by inducing senescence of aHSCs. Mechanistically, IL-10 attenuates CCL_4_-induced LF in rats by inducing aHSCs senescence via STAT3-p53 signaling pathway [32]. Similarly, elevated hepatic IL-22 levels in viral hepatitis patients can induce aHSCs senescence via STAT3-SOCS3-p53 signaling axis, thereby restricts LF in mice [33]. However, the role of senescent HSCs is dualistic: while senescent HSCs may directly inhibit fibrosis, they may also contribute to the fibrogenic microenvironment through the release of senescence-associated secretory phenotype (SASP) factors, which promote inflammation and fibrosis in neighboring cells [34]. The aforementioned studies indicate that the role of senescent HSCs in liver fibrosis is complex. However, our research findings reveal that senescent HSCs contributes to alleviating the progression of liver fibrosis.

MSCs are a type of adult stem cells with multidirectional differentiation potential, capable of exerting antifibrotic effects through mechanisms such as immunoregulation, secretion of antifibrotic cytokines, and regulation of HSCs activation[35]. For instance, bone marrow-derived MSCs (BM-MSCs) can inhibit HSCs activation by promoting the polarization of M1 macrophages to M2 macrophages, thereby alleviating liver fibrosis (LF) [36]. Umbilical cord-derived MSCs (UC-MSCs) and their conditioned media can induce HSCs apoptosis and inhibit their activation [37]. In contrast, ADSCs, as a subset of MSCs, not only share similar characteristics such as multidirectional differentiation potential, immunoregulatory functions, and the ability to secrete bioactive factors, but also exhibit unique advantages: [1] Easy accessibility: ADSCs can be abundantly obtained from adipose tissue through minimally invasive surgeries (such as liposuction) with minimal donor trauma [38]. [2] Robust proliferative capacity: ADSCs demonstrate superior proliferative ability *in vitro*, making them more suitable for research and clinical applications [39]. These advantages highlight the unique potential of ADSCs in disease treatment. While numerous studies have demonstrated the therapeutic effects of MSCs on liver fibrosis caused by various etiologies, there is currently no evidence suggesting that MSCs alleviate liver fibrosis by inducing HSCs senescence [40]. Consistent with previous findings, our study confirm that ADSCs transplantation effectively reduces HSCs activation levels, thereby alleviating LF in two distinct models. More importantly, we demonstrate for the first time that ADSCs transplantation can induce senescence in aHSCs, further elucidating a novel mechanism of MSCs in combating LF.

Despite the immense potential of cell-based therapeutic strategies in the treatment of LF, their clinical application still faces numerous challenges. Firstly, the engraftment efficiency of ADSCs transplantation is influenced by various factors, including the route of administration and microenvironmental compatibility. In our study, we employed two administration routes: tail vein injection and portal vein injection. Due to severe peritoneal adhesions in the Em group, which made portal vein injection technically challenging, we chose tail vein injection for ADSCs delivery. For the CCl_4_ model, we opted for portal vein injection of ADSCs. The number of transplanted ADSCs and the sampling time points were consistent across both models. Although studies have suggested that different administration routes may affect the therapeutic efficacy of ADSCs, portal vein injection has shown certain advantages in enhancing the hepatic homing of ADSCs [41–43]. However, our study demonstrated that ADSCs transplantation through either route could improve LF, with no statistically significant difference observed between the two methods. Additionally, immune-mediated cell clearance is another critical factor affecting the therapeutic efficacy of ADSCs. Although ADSCs exhibit low immunogenicity, a strong immune response microenvironment in the host (e.g., insufficient immunosuppression or severe infection) may still trigger ADSCs rejection, leading to reduced cell survival and diminished therapeutic effects. Our study confirmed that a robust inflammatory response exerts cytotoxic effects on transplanted ADSCs. Through preliminary experiments, we identified a critical time window for ADSCs transplantation—early intervention yields optimal therapeutic outcomes for LF caused by *E. multilocularis* infection [26]. In contrast, delayed intervention may result in non-specific immune-mediated clearance of ADSCs due to the intense inflammatory response surrounding the lesion. To address this limitation, we are currently developing nanoparticle-based delivery systems to protect ADSCs from immune attacks.

In our study, many unresolved issues and questions remain, requiring further research and clarification. Our research suggests that ADSCs-induced senescence of aHSCs could be an effective strategy against LF. However, further studies are needed to explore interventions targeting the pathways that induce aHSCs senescence, aiming to determine the feasibility of this treatment approach. For instance, Zhang et al. [44] reported that TWEAK promotes p53 deacetylation, which affects the senescence of human HSCs. Additionally, curcumin inhibits TGF-β signaling by activating peroxisome proliferator-activated receptor γ (PPARγ) and suppressing the gene expression of TGF-β receptors. The activation of PPARγ plays a crucial role in inhibiting HSCs activation and ECM production [45]. Similarly, we demonstrated that ADSCs transplantation upregulates PPARγ expression in aHSCs, suggesting that PPARγ could be a key target for inducing aHSCs senescence. Therefore, we hypothesize that exploring the mechanism by which ADSCs transplantation promotes aHSCs senescence through targeting key senescence-associated markers is both feasible and meaningful. Since HSCs account for a relatively small proportion of the total liver cell population, even under fibrotic conditions, Western blotting at the whole-liver level cannot effectively detect HSC-specific changes. Therefore, our study primarily utilized immunohistochemical methods to evaluate senescence markers in HSCs, thereby excluding potential interference from other senescent cell types. In future research, we plan to delve deeper into this area. Additionally, previous studies have revealed the positive role of ADSCs in resisting cellular senescence. Specifically, extracellular vesicles (EVs) derived from ADSCs mitigate endothelial cell senescence by inhibiting miR-674-5p and upregulating the expression of C1q/TNF-related protein 9 (CTRP9), thereby improving endothelial dysfunction [46]. Additionally, ADSCs transplantation inhibits the PI3K/Akt/mTOR signaling pathway, leading to reduced apoptosis and senescence of granulosa cells, increased follicle counts, and enhanced hormone secretion, effectively alleviating chemotherapy-induced premature ovarian failure (POF) [47]. In our study, we observed distinct cellular responses to ADSCs treatment in models of CCl_4_-induced liver injury and *E. multilocularis* infection. Notably, ADSCs therapy not only induced senescence in aHSCs but also attenuated hepatocyte senescence. This cell type-specific regulation may result from differences in receptor expression patterns among various cell types and the multifaceted paracrine signaling of ADSCs, although the specific mechanisms still require further investigation. Lastly, the safety and long-term effects of ADSCs therapy require further validation. Although ADSCs have demonstrated promising antifibrotic effects *in vitro* and in animal experiments, their potential tumorigenic risk, along with the fate and functional stability of the cells post-transplantation, requires in-depth investigation [4,229]. For example, whether ADSCs undergo abnormal differentiation or malignant transformation *in vivo*, and whether the factors they secrete adversely affect host tissues, remain to be further elucidated. Moreover, issues such as the large-scale preparation of ADSCs, quality control, and standardized transplantation protocols remain key challenges that need to be addressed in future clinical translation. Future research should focus on optimizing transplantation strategies, exploring the regulatory mechanisms of senescent cells, and systematically evaluating the long-term safety and efficacy of ADSCs therapy to facilitate its clinical translation.

## Conclusions

**Fig 7:**
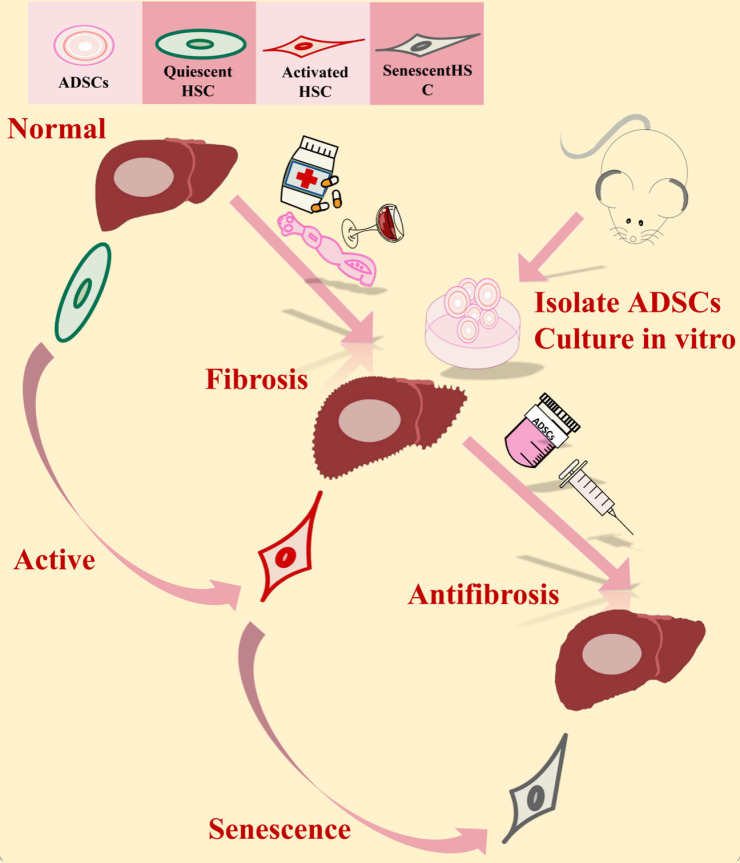
Schematic diagram of ADSCs therapeutic role in liver fibrosis of different etiologies by inducing aHSCs senescence.

Our study comprehensively elucidated the effectiveness of ADSCs transplantation in mitigating LF, regardless of its etiology. The key finding is the induction of cellular senescence in aHSCs by ADSCs transplantation, which serves as a central mechanism for reducing collagen deposition and fibrosis progression. The consistency of these results across both chemical (CCl_4_-induced) and parasitic (*E. multilocularis* infection) models of LF underscores the potential for a pan-fibrotic therapeutic approach (Fig 7). The present study enriches the understanding of ADSCs-mediated antifibrotic mechanisms and holds promise for the development of novel therapies to improve clinical outcomes for patients with fibrosis-related diseases.

## Methods

The study followed the ARRIVE guidelines.

### Ethics statement

The utilization of animals for research was approved by the Institutional Animal Care and Use Committee as well as the Ethical Committee of the First Affiliated Hospital of Xinjiang Medical University (Approval No. K202110-18); the study presents its results in compliance with the ARRIVE criteria.

### Animal experiments

Thirty female C57BL/6J mice (weighing 18–20 g; sourced from Beijing Vital River Laboratory Animal Technology Co. Ltd, Beijing, China) were randomly allocated into six experimental groups (n = 5/group): Olive oil, CCl_4_, CCl_4_ + ADSCs, Sham, Em, and Em + ADSCs. Mice were habituated at 22°C ± 2°C, with a relative humidity of 50% ± 10% and a 12-h light/dark cycle to simulate natural circadian rhythms. An acclimatization period of one week was observed before initiating experimental interventions, in accordance with ARRIVE guidelines.

### Establishment of *E. multilocularis* infection models

Both Em and the Em + ADSCs groups were infected with protoscoleces (PSCs) suspended in normal saline through the hepatic portal vein (HPV), whereas the Sham group received an equivalent normal saline volume as previously described [26]. PSCs were isolated from intraperitoneal lesions in Mongolian gerbils, meticulously rinsed with phosphate-buffered saline (PBS), counted microscopically, and diluted to a concentration of 3000 PSCs per 200 µL of saline prior to injection. The experimental models were anesthetized with isoflurane and subsequently administered PSCs or 200 μL of normal saline through the HPV, respectively.

### Establishment of CCl_4_ fibrosis models

Mice in the CCl_4_ group and the CCl_4_ + ADSCs group were administered intraperitoneal injections of 10% CCl_4_ at a dosage of 5 μL/g body weight, twice weekly for six weeks (Shanghai Macklin Biochemical Technology Co., Ltd, China). Following a previously established protocol, mice in the Olive oil group were administered an equivalent olive oil volume (Shanghai Yuanye Bio-Technology Co., Ltd., China) [48].

### Isolation and characterization of ADSCs

ADSCs were isolated and characterized using the following protocol. Briefly, inguinal adipose tissues were aseptically harvested from 6-8-week-old C57BL/6 mice for ADSCs isolation. The adipose tissues were minced into small fragments and enzymatically digested using 200 U/mL collagenase type I solution (Worthington, USA) at 37°C for 30 minutes.

The resulting cell suspension was then centrifuged and resuspended in α-MEM complete medium supplemented with 10% fetal bovine serum (FBS; both from Gibco, USA), 2 mM L-glutamine, and 100 U/mL penicillin-streptomycin (both from Hyclone, USA). After washing with PBS containing 1000 U/mL penicillin-streptomycin, the cells were plated and maintained at 37°C in a 5% CO2 humidified atmosphere. Following 48 h of initial adhesion, the culture medium was refreshed every 2–3 days until cells reached 85% confluence. Subsequent passages were performed at a seeding density of 4 × 10^4 cells/mL.

For differentiation studies, third-passage ADSCs were seeded at a density of 4 × 10^3 cells/cm² and allowed to reach 70% confluence before induction. Osteogenic differentiation was initiated using osteogenic induction medium, and the process was maintained for approximately three weeks until visible calcium deposition was observed. The cells were then fixed with 4% (v/v) paraformaldehyde and stained with Alizarin Red S to visualize calcium nodules, which were quantified using a Leica DM6000B microscope (Germany). Similarly, adipogenic differentiation was induced using specific induction medium for approximately four weeks until more than 70% of cells exhibited lipid droplet formation. The adipocytes were fixed with 4% (v/v) paraformaldehyde and stained with Oil Red O for lipid droplet visualization under the same microscope system (S3 Fig). Both differentiation protocols were performed according to previously established methods [26].

### Flow cytometry analysis

Aiming to verify the identity of ADSCs, flow cytometry (FACS AriaII, BD, USA) was employed to assess the cell surface marker expression as previously described [26]. The third-passage ADSCs were digested and resuspended in 100 μL of PBS. Here, we incubated 1 × 10^6 cells with the fluorochrome-conjugated antibody for 30 minutes at room temperature in the dark. S1 Table lists the antibodies used.

### ADSCs transplantation experiments

Third-passag ADSCs were digested and resuspended in normal saline at a 2.5 × 10^5 cells/100 μL density. After six weeks of CCl_4_ modeling and five months of *E. multilocularis* infection modeling, the CCl_4_ + ADSCs group received ADSCs via HPV injection, while the Em + ADSCs group received ADSCs injection, with both groups receiving 100 µL containing 2.5 × 10^5 ADSCs per mouse. Mice in other groups were administered an equivalent volume of normal saline.

### Histopathological and Immunohistochemical analysis

Liver samples were collected for histological examination and fixed in 4% paraformaldehyde for 48 hours, following fixation, the samples were dehydrated, embedded in paraffin, and sectioned at 4 μm thickness, as previously described [26]. To assess liver damage, the sections were stained with H&E. The extent of LF was evaluated using Sirius Red staining, with the positively stained areas quantified to determine fibrosis levels and statistically compared among groups. For immunohistochemical analysis, paraffin-embedded sections were deparaffinized, rehydrated, and subjected to antigen retrieval. The sections were then incubated overnight at 4°C with primary antibodies (S2 Table), followed by a 2 hours incubation with corresponding secondary antibodies at room temperature. Sections were analyzed under a light microscope (Olympus, Japan) and quantitatively evaluated using Image-Pro Plus software (version 6.0.0.260, Media Cybernetics, USA). Positive areas of Sirius Red staining and α-SMA were measured to determine differences in each group. Both p16 and p21-positive cells were counted in three random fields under a low-power field (LPF) to determine differences in each group.

### SA-β-Gal staining

Tissues were fixed in 4% paraformaldehyde fixation for 4 hours, followed by overnight dehydration in 30% sucrose solution at room temperature. Tissues were prepared as frozen sections of 5 µm thickness and maintained at –20°C before use. For cellular staining, cells were washed with PBS after the medium medium was aspirated prior to staining. Both tissue sections and cultured cells were processed for SA-β-Gal staining according to the manufacturer’s protocol (Beyotime Biotechnology, China) [49]. Staining results were imaged using a light microscope (Olympus, Japan). SA-β-Gal-positive cells were counted in three random fields under a low-power field (LPF) to determine differences in each group.

### Immunofluorescence analysis

In order to conduct the co-localization investigation, immunofluorescence was utilized, as previously described [50]. After rewarming at room temperature, frozen sections were fixed with 4% paraformaldehyde for 15 minutes, rinsed with PBS, permeabilized with 0.1% Triton-100 for 15 minutes, blocked with 5% bovine serum albumin (BSA) for 30 minutes, and then incubated at 4°C overnight with the primary antibody (S3 Table) that had been diluted with the blocking solution. For 2 hours, the nuclei were stained with DAPI after the secondary antibody and incubated at room temperature in the dark. Confocal microscopy (Leica SP8, Germany) was used to take photographs of the immunofluorescence staining findings.

### *E. multilocularis* antigen preparation and *in vitro* stimulation

The *E. multilocularis* PSCs antigen (EmP) was acquired a previously established method [51]. Briefly, fresh PSCs derived from intraperitoneal lesions maintained in Mongolian gerbils were placed into Eppendorf tubes, and 500 µL of PBS along with 2–3 sterile 4 mm stainless steel beads were added. The tubes were then placed in a pre-cooled tissue homogenizer (KZ-III-96, Servicebio, China) and homogenized for 2–3 minutes at 60 Hz. After overnight incubation on a shaker at 4°C, the samples were centrifuged at 13,000 rpm for 10 minutes at 4°C. The protein content in the collected supernatant was quantified. The supernatant was then filtered through a 0.22 µm sterile filter and aliquoted for storage at –80°C before use.

### Cell culture and co-culture experiment

The mouse HSC line JS1 (OTWO biotech, Shenzhen, China) was cultured in DMEM medium containing 10% fetal bovine serum (FBS; both from Gibco, USA) at 37°C in a 5% CO_2_ environment. For the co-culture experiment, JS1 cells were seeded at a 5 × 10^4/cm^2^/well density and divided into the following groups: Control, TGF-β, EmP, TGF-β + ADSCs, and EmP + ADSCs groups. Upon reaching 60%-70% confluence, only the culture medium was refreshed in the Control group. For both the TGF-β and TGF-β + ADSCs groups, 15 ng/mL of recombinant TGF-β protein was added. Meanwhile, in the EmP group and the EmP + ADSCs group, 60 µg/mL of EmP was added. Transwell inserts (pore size of 0.4 µm) seeded with 1 × 10^5 ADSCs were added as the upper layer in the culture wells of both the TGF-β + ADSCs and EmP + ADSCs groups. Cellular proteins from all groups were collected and extracted 24 hours after respective treatments [52].

### Western blot analyses

Cellular proteins were extracted using RIPA lysis buffer containing a protease inhibitor mixture (Invitrogen). Protein concentrations were quantified using the BCA Protein Assay Kit (23225, Thermo Fisher Scientific, USA). Protein lysate aliquots (20 μg) were separated by 10% SDS-PAGE and transferred onto 0.45 μm PVDF membranes (Millipore). The membranes were blocked in 5% skim milk in TBST (pH 7.6) for 2 hours at room temperature and then incubated overnight at 4°C with primary antibodies (S4 Table). Protein expression levels were normalized to GAPDH using grayscale analysis in ImageJ software (version 1.51, National Institutes of Health, USA), and the data were presented as mean ± standard deviation.

### Statistical analysis

Data analysis and processing were performed using GraphPad PRISM 9 software (version 9.5.0, La Jolla, CA, USA). The statistical significance between two groups was determined using an unpaired t-test, and comparisons among three or more groups were performed using one-way ANOVA. Data are reported as mean ± SD, with statistical significance set at p < 0.05: *p < 0.05, **p < 0.01, ***p < 0.001, ****p < 0.0001

## Supporting information

S1 Fig Gross images of livers from different experimental groups in mice. (TIF)

S2 Fig Detection of Alanine aminotransferase and aspartate aminotransferase in liver samples from various groups of mice. (a) Changes of serum ALT and AST in CCl_4_ model after transplantation of ADSCs. (b) Changes of serum ALT and AST in *E. multilocularis* infection model after transplantation of ADSCs. Data are presented as mean ± SD, *p* ＜ 0.05 was the threshold for significance. * *p* < 0.05, ** *p* < 0.01, *** *p* < 0.001, **** *p* < 0.0001.
(TIF)

S3 Fig Characterization of ADSCs. (a) Morphologies of ADSCs at the 3rd passage. (b) Adipogenic differentiation of ADSCs at the 3rd passage. (c) Osteogenic differentiation of ADSCs at the 3rd passage. (d) Flow cytometric characterization of ADSCs. The experiment was conducted with three replicates.
(TIF)

S1 Table Antibodies information used in flow cytometry analysis. (DOCX)

S2 Table Antibodies information used in immunohistochemical analysis. (DOCX)

S3 Table Antibodies information used in Immunofluorescence analysis. (DOCX)

S4 Table Antibodies information used in Western blot analysis. (DOCX)

S1 Data Raw data.
(XLSX)
