# Supplementary figures and images for: ADSCs attenuate Liver fibrosis via inducing HSC senescence: validation in dual-etiology models
Source: PLoS Negl Trop Dis. 2025 May 22;19(5):e0013094. doi: 10.1371/journal.pntd.0013094 (PMC12148229; doi:10.1371/journal.pntd.0013094)

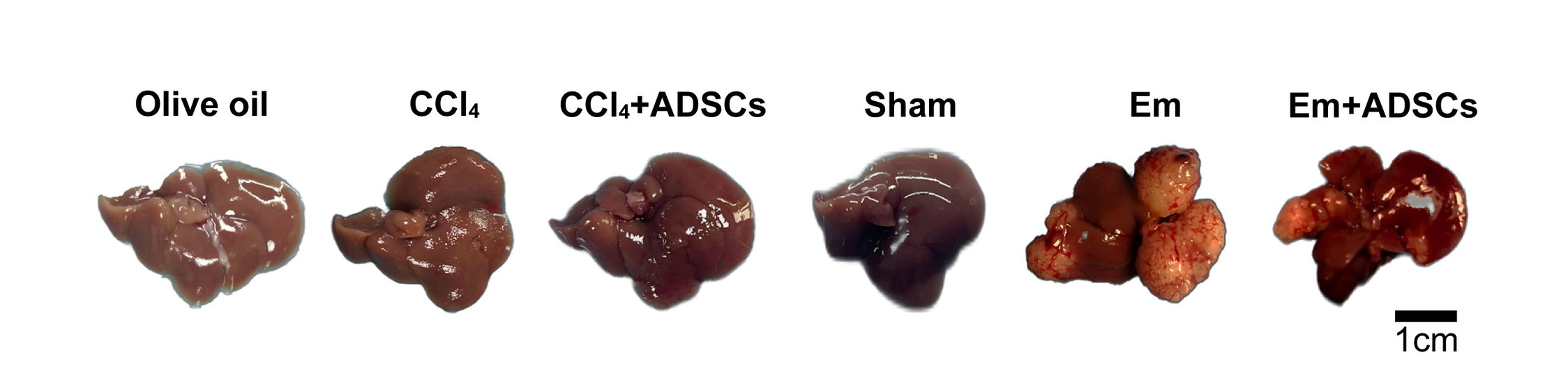

Supplement: S1 Fig — (TIF) [file pntd.0013094.s001.tif]

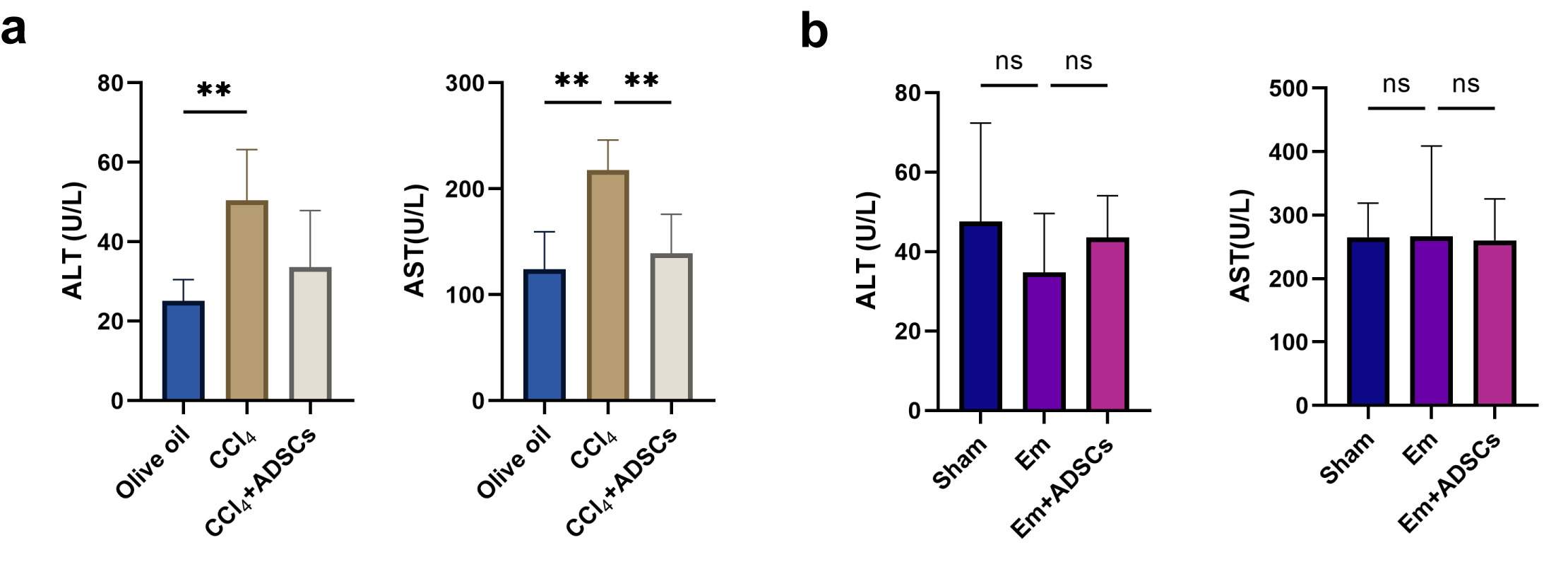

Supplement: S2 Fig — (b) Changes of serum ALT and AST in E. multilocularis infection model after transplantation of ADSCs. Data are presented as mean ± SD, p ＜ 0.05 was the threshold for significance. * p < 0.05, ** p < 0.01, *** p < 0.001, **** p < 0.0001. (TIF) [file pntd.0013094.s002.tif]

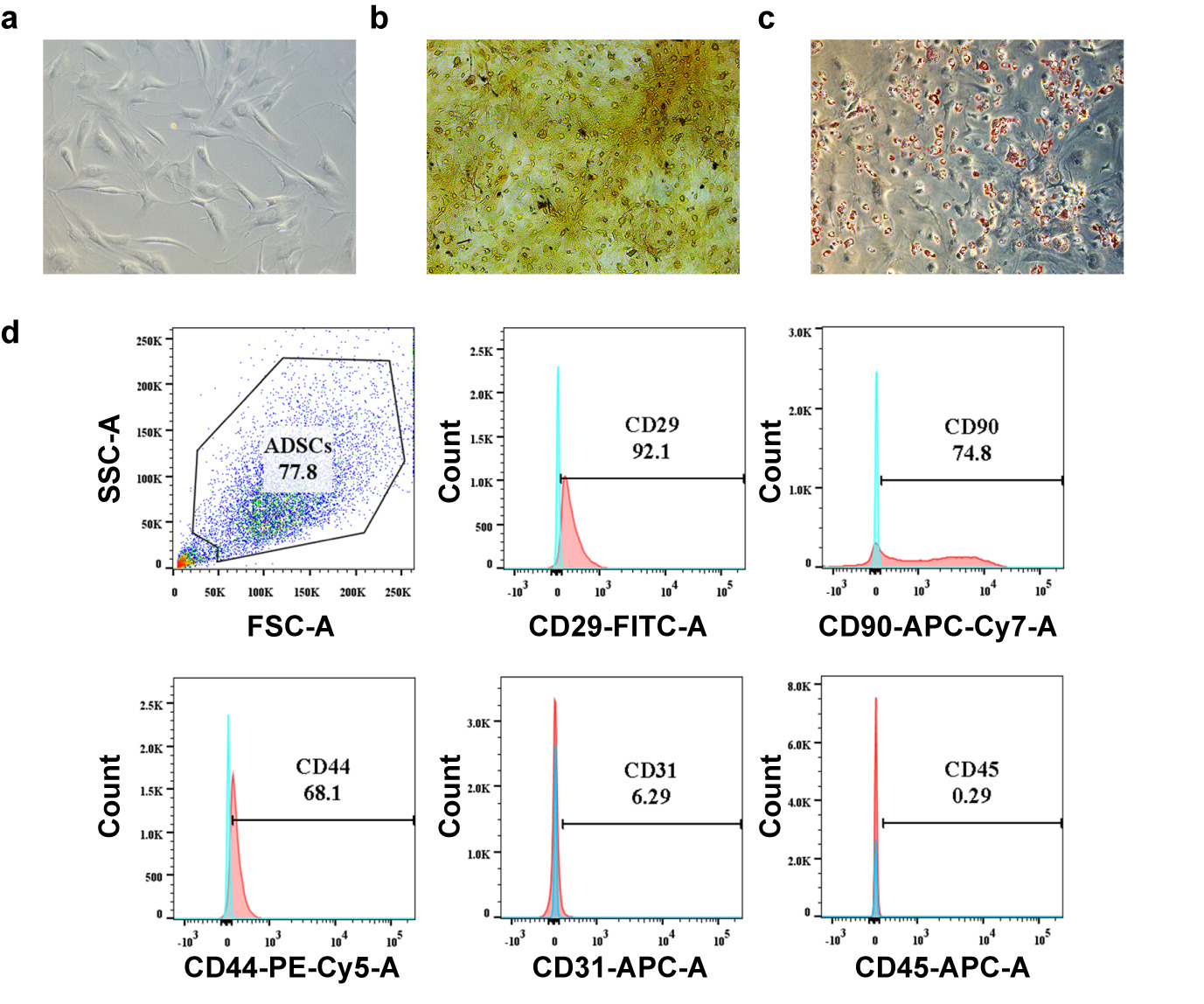

Supplement: S3 Fig — (a) Morphologies of ADSCs at the 3rd passage. (b) Adipogenic differentiation of ADSCs at the 3rd passage. (c) Osteogenic differentiation of ADSCs at the 3rd passage. (d) Flow cytometric characterization of ADSCs. The experiment was conducted with three replicates. (TIF) [file pntd.0013094.s003.tif]
