# Supplementary material for: ADSCs attenuate Liver fibrosis via inducing HSC senescence: validation in dual-etiology models
Source: PLoS Negl Trop Dis. 2025 May 22;19(5):e0013094. doi: 10.1371/journal.pntd.0013094 (PMC12148229; doi:10.1371/journal.pntd.0013094)
Supplement: S1 Table — (DOCX) [file pntd.0013094.s004.docx]

**S1 Table. Antibodies information used in flow cytometry analysis**

| **Name** | **Supplier** | **Catalog number** | **Clone number** | **Ratio** |
| --- | --- | --- | --- | --- |
| Rat Anti-Mouse CD31 | BD, US | 551262 | MEC 13.3 | 1:10 |
| Rat Anti-Mouse CD45 | BD, US | 559864 | 30-F11 | 1:10 |
| Hamster Anti-mouse CD29 | Biolegend, US | 102205 | HMβ1-1 | 1:40 |
| Rat Anti-Mouse CD44 | Biolegend, US | 103009 | IM7 | 1:10 |
| Rat Anti-Mouse CD90 | BD, US | 561641 | 53-2.1 | 1:40 |
