# Supplementary material for: ADSCs attenuate Liver fibrosis via inducing HSC senescence: validation in dual-etiology models
Source: PLoS Negl Trop Dis. 2025 May 22;19(5):e0013094. doi: 10.1371/journal.pntd.0013094 (PMC12148229; doi:10.1371/journal.pntd.0013094)
Supplement: S2 Table — (DOCX) [file pntd.0013094.s005.docx]

**S2 Table. Antibodies information used in immunohistochemical analysis**

| **Name** | **Supplier** | **Catalog number** | **Ratio** |
| --- | --- | --- | --- |
| Anti-α-SMA | Abcam, US | ab124964 | 1:1000 |
| Anti-p21 | Abcam, US | ab188224 | 1:1000 |
| Anti-P16 | Abcam, US | ab189034 | 1:1000 |
| Goat Anti-Rabbit  IgG - H&L (HRP) | Abcam, US | ab6013 | 1:1000 |
