# Supplementary material for: ADSCs attenuate Liver fibrosis via inducing HSC senescence: validation in dual-etiology models
Source: PLoS Negl Trop Dis. 2025 May 22;19(5):e0013094. doi: 10.1371/journal.pntd.0013094 (PMC12148229; doi:10.1371/journal.pntd.0013094)
Supplement: S3 Table — (DOCX) [file pntd.0013094.s006.docx]

**S3 Table. Antibodies information used in Immunofluorescence analysis**

| Name | Supplier | Catalog number | Ratio |
| --- | --- | --- | --- |
| Anti-a-SMA | CST, US | 48938S | 1:200 |
| Anti-p21 | Abcam, US | ab188224 | 1:200 |
| IgG (H+L)488 Anti-Rabbit | CST, US | 4412S | 1:400 |
| IgG (H+L)594 Anti-Mouse | CST, US | 8890S | 1:400 |
