# Supplementary material for: ADSCs attenuate Liver fibrosis via inducing HSC senescence: validation in dual-etiology models
Source: PLoS Negl Trop Dis. 2025 May 22;19(5):e0013094. doi: 10.1371/journal.pntd.0013094 (PMC12148229; doi:10.1371/journal.pntd.0013094)
Supplement: S4 Table — (DOCX) [file pntd.0013094.s007.docx]

**S4 Table. Antibodies information used in Western blot analysis**

| Name | Supplier | Catalog number | Ratio |
| --- | --- | --- | --- |
| Anti-P21 | Abcam, US | ab188224 | 1:1000 |
| Anti-P16 | Abcam, US | ab189034 | 1:1000 |
| Anti-P53 | Santa Cruz, US | SC-126 | 1:200 |
| Anti-GAPDH | Abcam, US | ab181602 | 1:10000 |
| Anti-α-SMA | Abcam, US | ab124964 | 1:5000 |
| m-IgG κ BP-HRP | Santa Cruz, US | SC-516102 | 1:1000 |
| IgG H&L/HRP | Bioss, China | bs-0295G-HRP | 1:5000 |
